# Ambulance Services Attendance for Mental Health and Overdose Before and During COVID-19 in Canada and the United Kingdom: Interrupted Time Series Study

**DOI:** 10.2196/46029

**Published:** 2024-05-10

**Authors:** Graham Law, Rhiannon Cooper, Melissa Pirrie, Richard Ferron, Brent McLeod, Robert Spaight, A Niroshan Siriwardena, Gina Agarwal, UK Canada Emergency Calls Data Analysis and GEospatial Mapping (EDGE) Consortium

**Affiliations:** 1 Community and Health Research Unit School of Health and Social Care University of Lincoln Lincolnshire United Kingdom; 2 Department of Family Medicine McMaster University Hamilton, ON Canada; 3 Department of Health Research Methods, Evidence, and Impact McMaster University Hamilton, ON Canada; 4 Niagara Emergency Medical Services Niagara, ON Canada; 5 Hamilton Paramedic Service Hamilton, ON Canada; 6 East Midlands Ambulance Service NHS Trust Nottingham United Kingdom

**Keywords:** COVID-19, mental health, overdose, emergency medical services, administrative data, Canada, the United Kingdom, ambulance, sex, age, lockdown, pandemic planning, emergency service

## Abstract

**Background:**

The COVID-19 pandemic impacted mental health and health care systems worldwide.

**Objective:**

This study examined the COVID-19 pandemic’s impact on ambulance attendances for mental health and overdose, comparing similar regions in the United Kingdom and Canada that implemented different public health measures.

**Methods:**

An interrupted time series study of ambulance attendances was conducted for mental health and overdose in the United Kingdom (East Midlands region) and Canada (Hamilton and Niagara regions). Data were obtained from 182,497 ambulance attendance records for the study period of December 29, 2019, to August 1, 2020. Negative binomial regressions modeled the count of attendances per week per 100,000 population in the weeks leading up to the lockdown, the week the lockdown was initiated, and the weeks following the lockdown. Stratified analyses were conducted by sex and age.

**Results:**

Ambulance attendances for mental health and overdose had very small week-over-week increases prior to lockdown (United Kingdom: incidence rate ratio [IRR] 1.002, 95% CI 1.002-1.003 for mental health). However, substantial changes were observed at the time of lockdown; while there was a statistically significant drop in the rate of overdose attendances in the study regions of both countries (United Kingdom: IRR 0.573, 95% CI 0.518-0.635 and Canada: IRR 0.743, 95% CI 0.602-0.917), the rate of mental health attendances increased in the UK region only (United Kingdom: IRR 1.125, 95% CI 1.031-1.227 and Canada: IRR 0.922, 95% CI 0.794-1.071). Different trends were observed based on sex and age categories within and between study regions.

**Conclusions:**

The observed changes in ambulance attendances for mental health and overdose at the time of lockdown differed between the UK and Canada study regions. These results may inform future pandemic planning and further research on the public health measures that may explain observed regional differences.

## Introduction

As the SARS-CoV-2 (COVID-19) pandemic has spread across the globe, it presents a major threat to mental health in general and is related to alcohol and substance use [1-3]. Government responses, in the form of travel restrictions and economic support for the COVID-19 pandemic, vary greatly from country to country [4]. Sweeping border closures and strict public health measures were variably implemented by many governments in an effort to contain the virus. However, inconsistencies in government responses may have contributed to fear and uncertainty, while strict public health measures may lead to social isolation [5].

Anxiety, depression, poor sleep quality, and psychological distress are mental health symptoms that have increased since the start of the COVID-19 pandemic [6]. Stress and poor mental health are being exacerbated by the uncertain future of the pandemic, misinformation, and social isolation brought about by physical distancing measures [7,8]. Factors such as sex and age have also been identified as risk factors for experiencing negative mental health effects of the COVID-19 pandemic [6,9]. Being younger and identifying as female have both been linked to experiencing lower psychological well-being throughout the course of the pandemic. Relatedly, there are also reports of an increase in overdoses during the COVID-19 pandemic [10,11]. This may be due to changes in the availability of illicit drugs during lockdown [2], possibly resulting in a higher rate of overdoses when access was restored, and also challenges in physically accessing pharmaceutical therapies (eg, methadone) [2]. In addition, a systematic review in 2022 reported that mental health and overdose are consistently found to be associated across studies, although the causal mechanism remains unclear [12]. In the context of COVID-19, it has been reported that social isolation has put at risk the vulnerable population of substance users, increasing the strain on mental health and subsequently further increasing the probability of an overdose [2]. Therefore, mental health and overdose are 2 related, but different, outcomes that should be examined in tandem.

Currently, studies examining the relationship between mental health and COVID-19 rely on self-reported measures [6,9,13]. Other indicators of population mental health and overdoses are of interest, especially for policy and resource planning. Emergency medical service (EMS) data, such as attendances, have previously been used to understand public health trends, which can help planning and resource allocation [14].

Due to variation in population characteristics and the government response to COVID-19 between countries, differences may exist in mental health effects. Specifically, the United Kingdom and Canada are 2 countries that experienced differing national responses to the ongoing pandemic. A report by the chief public health officer of Canada found that self-perceived mental health status decreased during the COVID-19 pandemic when compared to 2018 and that overdoses were increasing [11]. A comparison of the mental health and overdose effects of COVID-19 between Canada and the United Kingdom is of interest, as population characteristics and EMS attendance differ. Understanding the need for other indicators of mental health and overdose besides self-reporting, EMS data will be used to elucidate the impact of COVID-19 on EMS attendances.

Little is known about the trend in attendances before and after lockdown, especially for mental health and overdoses internationally. This paper aims to compare differences in the trends and volumes of mental health and overdose attendances before and after lockdown between specific regions in Canada and the United Kingdom. Evidence suggests that differences exist between mental health and overdose effects between sexes and age groups [6,9]. Therefore, this study will examine and describe the number of mental health and overdose attendances to 911 or 999 EMS calls in regions of the United Kingdom and Canada according to population subgroups.

## Methods

### Study Design

We used an interrupted time series design, analyzing weekly ambulance attendances before and after the COVID-19 lockdown from regions of the United Kingdom (East Midlands) and Canada (Niagara and Hamilton, Ontario). The East Midlands Ambulance Service NHS Trust is a regional EMS that operates in the East Midlands of the United Kingdom. In Canada, 2 services were examined: Niagara Emergency Medical Services is a moderate-sized EMS that operates in the Niagara region of Ontario, Canada, and Hamilton Paramedic Service is a moderate-sized EMS in Hamilton, Ontario. We purposefully selected these regions in the United Kingdom and Canada for comparison since they have similar population densities and median household incomes, and it was feasible to obtain their EMS call records.

### Population

In Canada, the study population was those attended to by Niagara Emergency Medical Services and Hamilton Paramedic Service in Ontario. The population of Niagara and Hamilton was obtained from the 2021 Canadian Census Profile [15]. In the United Kingdom, the study population was the East Midlands region of England, which is serviced by East Midlands Ambulance Service NHS Trust. The population for the East Midlands region was obtained from the Office for National Statistics using the midyear population estimates [16] by age and sex.

### Ethical Considerations

In the United Kingdom, the study was given NHS favorable ethical opinion (reference 20/SC/0307) by the South Central—Berkshire B Research Ethics Committee and is listed on the Integrated Research Application System as 286198. Patient records for this study were anonymized by the ambulance service, and informed consent could not be feasibly obtained for this retrospective study of administrative data. In Canada, the study was reviewed by the Hamilton Integrated Research Ethics Board, and a waiver letter was issued since individual patient data were not released by the paramedic services, and only weekly aggregated administrative data for the entire region were provided for this study.

### Data Collection

#### Overview

The weekly number of ambulance attendances (Sunday to Saturday) was collected covering 2 adjacent time periods: the weeks leading up to the lockdown (December 29, 2019, until March 21, 2020, in the United Kingdom and March 14, 2020, in Canada) and the weeks during and following the lockdown (March 22, 2020 and March 15 in the United Kingdom and Canada, respectively, until August 1, 2020). The lockdown occurred on March 23, 2020, in the United Kingdom and March 15, 2020, in Canada.

#### Mental Health and Overdose

Ambulance call records for mental health and overdose were identified by the ambulance services through clinical impression (provisional diagnosis) codes recorded by ambulance staff when attending the patient. The UK and Canadian systems for assigning problem codes to paramedic-patient encounters are similar, though the actual codes available to select from are slightly different in each country. Work to map the 2 countries’ systems of paramedic coding is underway [17]. Where more than one problem code existed (Canada), data for all problem codes (primary, secondary, and final) were extracted. The mental health records were identified in Canada as clinical impression or problem code 45 (behavior or psychiatric) and in the United Kingdom as the clinical impressions “admission under mental health act,” “anxiety,” “attempted suicide,” “deliberate self-harm,” “depression,” “panic attack,” “psychosis,” and “other mental health.” For overdose, the records were identified in Canada as clinical impression or problem codes 81 (drug or alcohol overdose), 81.1 (opioid overdose), or 81.2 (alcohol intoxication) and in the United Kingdom as clinical impression “intentional drug overdose (mental health),” “accidental overdose or poisoning (medical),” “effects of alcohol,” and “query intoxicated (medical).”

#### Sex and Age Groups

Sex was recorded in Canada and the United Kingdom by the crew in attendance. Age was recorded from self-report or estimated by the crew in attendance where this was not possible. Age groups were used to categorize the data into 18 years and younger, 18-44 years, 45-65 years, and 65 years and older.

### Statistical Analysis

**Figure 1:**
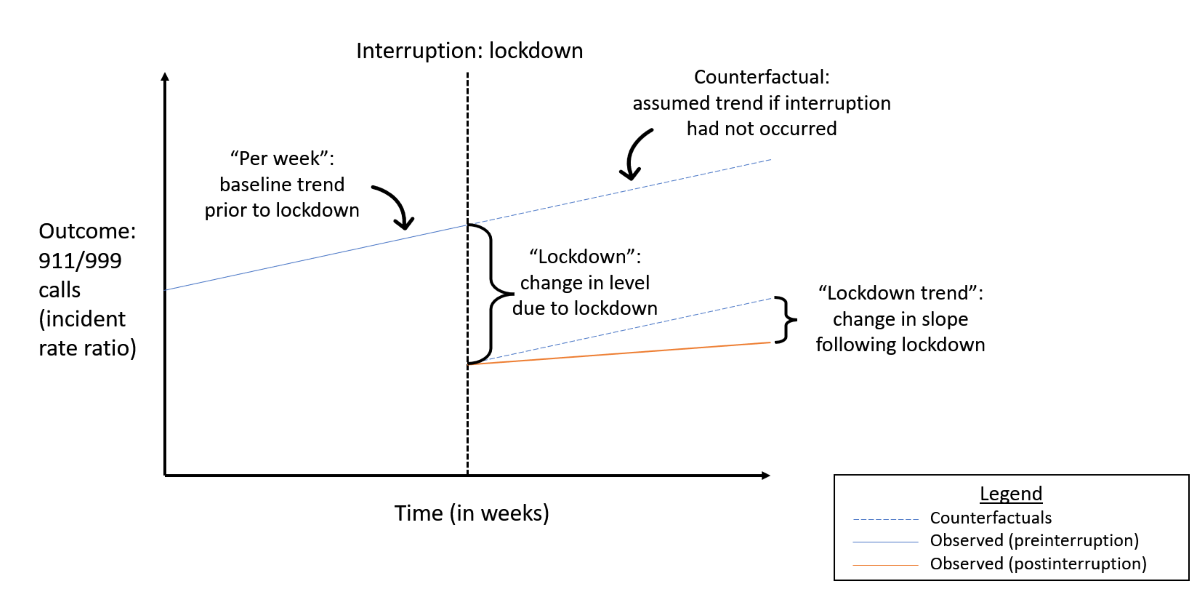
Interrupted time series study design used to model the change in weekly emergency medical service calls at the time of lockdown and the period following lockdown.

Using an interrupted time series approach (Figure 1), the number of weekly attendances was modeled as the outcome using negative binomial regression, and the results were reported as incidence rate ratios (IRRs). As shown in Figure 1, for each regression, the prelockdown trend of weekly calls was modeled (per week), and then a counterfactual scenario was imagined, where the trend in the data would have continued without the interruption. The counterfactual scenario provided a comparison for the evaluation of the impact of the lockdown by examining the change in level at the time of the lockdown (lockdown) and the change in the slope during lockdown (lockdown trend) relative to this counterfactual scenario.

Negative binomial regression was identified as the most appropriate model since there was overdispersion observed in the outcome variables. The models were fitted using time (in weeks relative to the lockdown start week), lockdown (a binary categorical variable of before lockdown and during lockdown), lockdown trend (time in weeks following lockdown, which indicates the slope change following lockdown), and seasonality (an adjustment made via the categorical variable of month) with an offset of total population size. All statistical analysis was conducted using R (R Foundation for Statistical Computing).

## Results

Table 1 shows there were 4.9 million people living in the area served by the ambulance service in the East Midlands region of the United Kingdom and 1.0 million in the Canadian regions of Hamilton and Niagara. The proportion of female individuals in each location was similar (n=2,457,905, 50.5% vs n=522,488, 51.2%), as were the proportions of individuals aged 65 years and older (n=902,947, 18.6% vs n=207,184, 20.5%). Household incomes and population densities were found to be similar, as well (Table 1). In the Canadian study regions, the ambulance services provided 1 vehicle per 14,776 persons, while in the UK study region, it was 1 per 9809 persons.

**Table 1:** Description of the UK and Canadian study regions, including population size by sex and age, geographic area, household income, and paramedic capacity.

			East Midlands region, United Kingdom (n=4,865,583)		Niagara and Hamilton regions, Canada (n=1,019,510)
**Sex, n (%)**					
	Female	2,457,905 (50.5)		522,488 (51.2)	
	Male	2,407,678 (49.5)		497,022 (48.8)	
**Age (years), n (%)**					
	<18	1,007,895 (20.7)		206,428 (20.4)	
	18-44	1,623,597 (33.4)		306,517 (30.4)	
	45-65	1,331,144 (27.4)		289,575 (28.7)	
	>65	902,947 (18.6)		207,184 (20.5)	
**Geographic and population description**					
	Area (km^2^)	16,640		3226	
	Population density (residents per square kilometer)	290		316	
	Adjusted household income (US $)^a^	$44,357^b^		$46,003^c^	
**Paramedic service description**					
	Peak number of vehicles staffed	496		69 (38 Niagara and 31 Hamilton)	
	Number of persons per vehicle	9809		14,776	
	Total number of crew staff	3200		745 (343 Niagara and 402 Hamilton)	

^a^Adjusted household income using the Organisation for Economic Co-operation and Development purchasing power parities [18] based on a standardized basket of goods and services for the United Kingdom (0.7) and Canada (1.3).

^b^The mean disposable income, according to the United Kingdom Office for National Statistics, 2016 census [19].

^c^The median after-tax income, according to Statistics Canada, 2016 census [20]; weighted median using the Hamilton and Niagara population totals from the same year.

During the study period, there were 182,947 attendances for mental health and overdose in the study regions (Table 2 and Table S1 in Multimedia Appendix 1), with an average of 1042.9 calls per week for mental health and 776.4 calls per week for overdose in the UK regions, and 232.5 calls per week for mental health and 139.1 calls per week for overdose in the Canadian regions. The interrupted time series analysis showed that in the time prior to the lockdown, attendances in the UK region for mental health had a small but statistically significant increasing rate across weeks, whereas the rate of these attendances was steady week-over-week in the Canadian regions. When the lockdown was initiated (the interruption in the time series) the rate of attendances for mental health in the UK region had a statistically significant increase and then remained stable with no statistically significant slope for the weeks during lockdown, while the Canadian regions saw no change when the lockdown was initiated (interruption) or in the slope during lockdown.

**Table 2:** Interrupted time series of weekly emergency medical service calls for mental health and overdose from January 2019 to July 2020, using negative binomial regression offset by the total population, for regions in the United Kingdom and Canada.^a^

Outcome and variable		East Midlands region, United Kingdom	Niagara and Hamilton regions, Canada
**Mental health**			
	Attendances, n	87,086	19,410
	Per week, IRR^b^ (95% CI)	*1.002 (1.002-1.003)* ^c^	0.999 (0.999-1.000)
	Lockdown, IRR (95% CI)	*1.125 (1.031-1.227)*	0.927 (0.802-1.073)
	Lockdown trend, IRR (95% CI)	0.998 (0.990-1.006)	1.003 (0.989-1.017)
**Overdose**			
	Attendances, n	64,832	11,619
	Per week, IRR (95% CI)	*1.002 (1.002-1.003)*	*1.002 (1.000-1.002)*
	Lockdown, IRR (95% CI)	*0.573 (0.518-0.635)*	*0.758 (0.615-0.936)*
	Lockdown trend, IRR (95% CI)	*1.031 (1.022-1.041)*	1.015 (0.994-1.035)

^a^Models have been adjusted for seasonality (each month as a variable).

^b^IRR: incidence rate ratio.

^c^IRRs statistically significant at *P*<.05 are indicated in italics format.

Overdose attendances had a similar small but statistically significant weekly increase in both the UK and Canadian regions, and both had statistically significant drops in the rate of overdose attendances when the lockdown was initiated. However, while the UK study region rebounded to having a statistically significant positive slope in attendance rate after lockdown, the Canadian regions maintained this lower level of attendances after lockdown with a statistically nonsignificant slope.

Table 3 shows model estimates for the rate of mental health attendance by sex and age categories across the study weeks (also see Table S2 in Multimedia Appendix 1). For the Canadian regions, although the full sample showed no statistically significant changes in mental health attendances, this subgroup analysis demonstrates that there were substantial changes for male individuals. Specifically, at the time of lockdown (interruption), there was a statistically significant decrease in the rate of mental health attendances for male individuals, which then rebounded with a positive slope during the lockdown period. In contrast, both male and female individuals in the UK region had an increase in the rate of mental health attendances when the lockdown was initiated and then held steady at that new level.

**Table 3:** For each sex and age category strata, an interrupted time series of weekly emergency medical service calls for mental health from January 2019 to July 2020, using negative binomial regression offset by the total population of subgroup, for regions in the United Kingdom and in Canada.^a^

Strata and variable			East Midlands region, United Kingdom	Niagara and Hamilton regions, Canada
**Sex**				
	**Female**			
		Attendances, n	50,098	10,829
		Per week, IRR^b^ (95% CI)	*1.002 (1.002-1.003)* ^c^	0.999 (0.999-1.000)
		Lockdown, IRR (95% CI)	*1.101 (1.007-1.203)*	1.012 (0.856-1.196)
		Lockdown trend, IRR (95% CI)	0.998 (0.990-1.007)	0.992 (0.976-1.008)
	**Male**			
		Attendances, n	36,515	8581
		Per week, IRR (95% CI)	*1.002 (1.001-1.003)*	0.999 (0.999-1.000)
		Lockdown, IRR (95% CI)	*1.163 (1.050-1.288)*	*0.830 (0.702-0.980)*
		Lockdown trend, IRR (95% CI)	0.998 (0.989-1.008)	*1.017 (1.001-1.033)*
**Age**				
	**<18 years**			
		Attendances, n	4239	1572
		Per week, IRR (95% CI)	*1.003 (1.002-1.004)*	0.999 (0.997-1.001)
		Lockdown, IRR (95% CI)	*0.723 (0.579-0.913)*	0.687 (0.451-1.038)
		Lockdown trend, IRR (95% CI)	*1.021 (0.999-1.042)*	1.024 (0.985-1.065)
	**18-44 years**			
		Attendances, n	39,648	10,382
		Per week, IRR (95% CI)	*1.003 (1.002-1.003)*	1.000 (0.999-1.001)
		Lockdown, IRR (95% CI)	1.097 (0.986-1.220)	0.969 (0.824-1.139)
		Lockdown trend, IRR (95% CI)	0.994 (0.985-1.004)	0.997 (0.982-1.012)
	**45-65 years**			
		Attendances, n	22,970	4764
		Per week, IRR (95% CI)	*1.002 (1.001-1.002)*	0.999 (0.998-1.001)
		Lockdown, IRR (95% CI)	*1.255 (1.123-1.403)*	0.905 (0.723-1.131)
		Lockdown trend, IRR (95% CI)	0.991 (0.981-1.002)	1.012 (0.991-1.012)
	**>65 years**			
		Attendances, n	20,229	2692
		Per week, IRR (95% CI)	*1.001 (1.000-1.002)*	0.999 (0.997-1.000)
		Lockdown, IRR (95% CI)	*1.118 (1.005-1.244)*	0.938 (0.721-1.215)
		Lockdown trend, IRR (95% CI)	*1.011 (1.001-1.021)*	1.002 (0.978-1.027)

^a^Models have been adjusted for seasonality (each month as a variable).

^b^IRR: incidence rate ratio.

^c^IRRs statistically significant at *P*<.05 are indicated in italics format.

While the Canadian regions showed no statistically significant changes in the rate of mental health attendances by age category, the UK region found that those younger than 18 years of age had a statistically significant decrease in mental health attendances when the lockdown was initiated, while those aged 45-65 years and 65 years and older had a statistically significant increase. In addition, for those aged 65 years and older, the rate of attendances for mental health continued to increase during the lockdown period.

Table 4 shows the attendances for overdose in each of the sex and age subgroups (also see Table S3 in Multimedia Appendix 1). Similar to what was observed for mental health attendances, in the Canadian Region, there was a statistically significant drop in attendances for overdose at the time the lockdown was initiated; however, for this outcome, there was also a statistically significant drop for those aged 18-44 years. In the United Kingdom, a consistent statistically significant decrease in attendances for overdose was observed among all age and sex subgroups, followed by a statistically significant positive slope week-over-week for all subgroups in the period during lockdown.

**Table 4:** For each sex and age category strata, an interrupted time series of weekly emergency medical service calls for overdose from January 2019 to July 2020 using negative binomial regression offset by the total population, for regions in the United Kingdom and in Canada.^a^

Strata and variable			East Midlands region, United Kingdom	Niagara and Hamilton regions, Canada
**Sex**				
	**Female**			
		Attendances, n	29,939	4646
		Per week, IRR^b^ (95% CI)	*1.003 (1.002-1.004)* ^c^	*1.002 (1.001-1.003)*
		Lockdown, IRR (95% CI)	*0.535 (0.474-0.604)*	0.906 (0.723 1.134)
		Lockdown trend, IRR (95% CI)	*1.036 (1.024-1.048)*	1.005 (0.984-1.027)
	**Male**			
		Attendances, n	34,516	6973
		Per week, IRR (95% CI)	*1.002 (1.001-1.002)*	*1.001 (1.000-1.002)*
		Lockdown, IRR (95% CI)	*0.612 (0.547-0.685)*	*0.674 (0.522-0.872)*
		Lockdown trend, IRR (95% CI)	*1.028 (1.017-1.039)*	1.021 (0.996-1.046)
**Age**				
	**<18 years**			
		Attendances, n	5385	630
		Per week, IRR (95% CI)	*1.003 (1.002-1.004)*	*0.997 (0.994-0.999)*
		Lockdown, IRR (95% CI)	*0.456 (0.369-0.564)*	0.845 (0.469-1.469)
		Lockdown trend, IRR (95% CI)	*1.051 (1.031-1.071)*	1.007 (0.954-1.006)
	**18-44 years**			
		Attendances, n	37,314	7645
		Per week, IRR (95% CI)	*1.002 (1.002-1.003)*	*1.002 (1.001-1.003)*
		Lockdown, IRR (95% CI)	*0.572 (0.505-0.647)*	*0.703 (0.560-0.882)*
		Lockdown trend, IRR (95% CI)	*1.030 (1.019-1.042)*	1.013 (0.991-1.035)
	**45-65 years**			
		Attendances, n	17,313	2826
		Per week, IRR (95% CI)	*1.002 (1.002-1.003)*	*1.003 (1.000-1.004)*
		Lockdown, IRR (95% CI)	*0.583 (0.518-0.656)*	0.883 (0.638-1.223)
		Lockdown trend, IRR (95% CI)	*1.031 (1.020-1.042)*	1.019 (0.988-1.051)
	**>65 years**			
		Attendances, n	4820	518
		Per week, IRR (95% CI)	*1.003 (1.002-1.004)*	1.000 (0.997-1.003)
		Lockdown, IRR (95% CI)	*0.700 (0.577-0.848)*	0.855 (0.467-1.511)
		Lockdown trend, IRR (95% CI)	*1.024 (1.007-1.042)*	0.996 (0.929-1.053)

^a^Models have been adjusted for seasonality (each month as a variable).

^b^IRR: incidence rate ratio.

^c^IRRs statistically significant at *P*<.05 are indicated in italics format.

## Discussion

### Principal Findings

Our study found that there were statistically significant differences in the effects of lockdown on mental health and overdose attendances when comparing the United Kingdom and Canada. A web-based cross-sectional survey in the United Kingdom, 4 weeks into lockdown, found that 5% of participants were positive for a common mental health disorder [21]. In Canada, a cross-sectional survey found women of working age were most affected by a mental health condition, while 54% of Canadians reported good mental health [11]. While these surveys are helpful for understanding the self-reported mental health of populations, using EMS data as an indicator of mental health status among populations provides a powerful approach to understanding complex social changes in a novel way.

Both Canada and the United Kingdom experienced major societal changes during 2020 caused by the COVID-19 pandemic and took different approaches to addressing this event, which may explain observed differences in rates of mental health and overdose attendances for specific sex and age categories. The United Kingdom entered the first lockdown on March 23, 2020, while Ontario (Canada) had already reached this stage on March 15, 2020. Schools, colleges, and universities closed, and a large majority of the working population worked from home or was funded to not work (known as furlough in the United Kingdom). Hospitality services closed, and citizens were requested to remain at home. Primary care moved to web-based and telephone-based consultations, and general practitioners met patients in person only when necessary for examination. International borders were closed in Canada but remained largely open in the United Kingdom.

Financial benefits and emergency funding also differed between countries. The Canada Emergency Response Benefit paid a gross of CAD $2000 (equivalent to just under £1200 sterling or US $1480) per month for those who had lost their jobs or were unable to work. The United Kingdom relied on the standard benefit system, known as Universal Credit, for those who lost their jobs. Those still employed, but unable to work, were given 80% of their gross pay, up to a maximum of £2500 per month (equivalent to just over CAD $4200 or US $3100). Fiscal stimulus packages varied, with Canada investing 18.6% of gross domestic product and the United Kingdom 17.8% [22]. Finances are known to be a major cause of stress and mental health issues.

A measure of comparison between countries that actually allows us to compare the different approaches holistically is that of the stringency index. It is a measure of governmental and public health orders that took effect during the pandemic [4], allowing global comparisons due to a scoring system attributing values to components of lockdown (such as stay-at-home orders, business, and facility openings). A comparison of the United Kingdom’s and Canada’s stringency indices (shown in Multimedia Appendix 2) showed that, in fact, the United Kingdom and Canada experienced similar levels of lockdown, despite different governmental policies and other local public health unit mandates. The EMS data allow for health-related differences between countries that may be influenced by societal changes that have become evident, while contrasting comparisons of overall indices of lockdown stringency can concurrently be examined.

### Mental Health and Overdose

This study found that prior to the pandemic lockdown, overall the rate of mental health presentations was holding constant in Canada, and there was a very small increase over time in the United Kingdom. When the lockdown was initiated, a difference in EMS attendance was seen between the countries; in the United Kingdom, nonoverdose attendance for mental health increased substantially, while in Canada, there was no change. These observed differences between Canada and the United Kingdom may, in fact, be due to the differences in the governmental lockdowns and orders. In particular, health care workers have experienced a high prevalence of anxiety, depression, and insomnia. This may be due to several factors, including fear of infection and the overwhelming influx of new information pertaining to caring for patients with COVID-19 [23-25].

Both countries also showed similar rises in rates of attendances for overdoses prior to lockdown. The immediate effect of the lockdown was similar, with a large reduction in attendances for overdose in both the United Kingdom and Canada, although the rate was increasing again during the lockdown period in the United Kingdom. The level of overdoses may be impacted by not only mental health stressors but also other societal changes resulting from the pandemic. Where the availability of drugs and alcohol becomes reduced and supplies dwindle due to a lack of prescribing or a lack of street drugs, overdose may become more likely in the subsequent period, as tolerance to these substances may have decreased.

### Differences by Sex

In the UK study region, there were no sex differences for mental health and overdose attendances; both sexes had similar increases for mental health but decreases for overdose. However, in the Canadian study regions, there was a statistically significant decrease in both mental health and overdose attendances for male individuals, while there was no statistically significant change for female individuals. The reasons for these different trends among the sex strata within and between the UK and Canadian study regions are likely to be extremely complex and multifactorial, and therefore difficult to tease out in this paper without further study.

### Differences by Age

There has been debate about the impact of lockdown on children and young adults [26]. Schools closed in both the United Kingdom and Canada, and school-age children were taught by parents or guardians. In the UK study region, the week-over-week rate of mental health attendances was increasing in a similar manner for all age categories in the period before lockdown, but at the time of lockdown, there was a substantial drop in the rate of attendances among the individuals younger than 18-years of age, whereas there was an increase among the individuals aged 45-65 years and those older than 65 years. In contrast, in the Canadian study regions, there were no statistically significant changes in the rate of mental health attendances at the time the lockdown was initiated for any of the age category strata. For rates of overdose attendances, there was no noticeable effect demonstrated by different age group strata in Canada or the United Kingdom.

In the United Kingdom, examinations were canceled for the General Certificate of Secondary Education (16-year-olds) and A-levels (18-year-olds), which may have reduced stress. In addition, there may have been decreased stress due to learning at home, without the need to attend school in person, with less exposure to school-related stressors. This is supported by existing literature describing how adolescents with existing mental health problems pre-COVID-19 had less during the pandemic [27]. In Canada, the education system has no equivalent to the General Certificate of Secondary Education and A-level examinations; therefore, pandemic schooling-related effects would have been different and may not have been as pronounced, resulting in no statistically significant mental health changes pre-COVID-19 compared to during the pandemic.

In both the United Kingdom and Canada, there was considerable distress caused by the volume of vulnerable people in care homes and the speed of government responses. This was seen by the number of deaths among care-home residents [28] over the period from March 20, 2020, to January 15, 2021. In the United Kingdom, 33% (n=30,851) of COVID-19–related deaths were among people residing in care homes. In Canada, long-term care home residents accounted for 59% (n=11,114) of COVID-19–related fatalities. As a result of these problems, care home residents were subject to significant restrictions, leading to reduced access to medical care and isolation-related stress. Although it could be expected that the older age groups (45-55 years and 65 years and older) would worry about income, poor COVID-19 outcomes due to higher rates of chronic diseases [29], and about family and parents in care homes (United Kingdom) or long-term care facilities (Canada), these age groups had a statistically significant increase in mental health–related calls in the United Kingdom but not in Canada. Other literature from the United Kingdom demonstrates worse mental health in age groups younger than 35 years; however, the data were from a cross-sectional survey during lockdown [21]. These differences might be explained by behavioral differences in the reasons for which it was deemed appropriate to call EMS in each country, by less stress overall in one country versus the other, or by differences in the data collection method used to determine mental health problems (calls to EMS vs self-reported survey). However, further research is needed to understand these differences, which are beyond the scope of this paper.

### Strengths and Limitations

This study compares routinely collected observational data from 2 Western countries. Data were collected primarily for routine clinical recording, monitoring, and clinical auditing rather than for epidemiological research. These data have nevertheless allowed a careful examination of the ambulance response to mental health and overdose emergencies experienced by the populations, using data that are not self-reported. It should be noted that responses to mental health in both the United Kingdom and Canada tend to have similar patient pathways. Both countries have a national health system, in which health care is freely available to all; therefore, the pathways open to patients are to call 911/999 to visit a primary care doctor or facility where they can receive emergency or urgent care.

There will be confounders that may explain some of the predictions made by the statistical models. Age and sex were adjusted for, but data were not available to adjust for ethnicity or socioeconomic status. In the United Kingdom and Canada, there have been clear differences in the rates of COVID-19 between different ethnic groups and levels of affluence. These differences are also likely to contribute to differences in rates of mental illness and requirements for mental health support.

There were differences in the data collected between each country, and efforts were made to adjust for these underlying coding differences. Intentionality was not used to define overdose. Clinical impression (ambulance provisional diagnosis) codes were not entirely comparable between the 2 countries. In Canada, impression code 81 (drug or alcohol overdose) is captured by the 4 most relevant codes in the United Kingdom. There will be cases missed and documented as “other medical problem” or “acute behavioral disturbance,” for example, but that broadens the category too wide. The agreement was achieved by extracting comparable codes through discussion.

### Conclusions

The EMS attendance data have demonstrated varying impacts of the COVID-19 pandemic on mental health and overdose between the study regions in the United Kingdom and Canada. While the countries implemented similar degrees of lockdowns and public health measures, as seen with the stringency index, there were differences in the specific measures taken; these differences may explain the divergence in mental health attendances between the regions. In contrast, overdose attendances followed similar patterns between the study regions. Future research is needed to explore the mechanisms behind these observed trends in EMS attendances following the lockdown and to examine whether the countries return to having similar rates of attendances over time as they did prior to the lockdown.

## Appendix

Multimedia Appendix 1 Results tables.

Multimedia Appendix 2 Stringency index for the 2 study regions from January to July 2020.

## Abbreviations

EMS: emergency medical service
IRR: incidence rate ratio

## Data Availability

The data sets analyzed during this study are not publicly available due to restrictions by the paramedic and ambulance services but are available from the corresponding author on reasonable request.
