# Supplementary material for: Ambulance Services Attendance for Mental Health and Overdose Before and During COVID-19 in Canada and the United Kingdom: Interrupted Time Series Study
Source: JMIR Public Health Surveill. 2024 May 10;10:e46029. doi: 10.2196/46029 (PMC11090162; doi:10.2196/46029)
Supplement: Multimedia Appendix 1 [file publichealth_v10i1e46029_app1.pdf]

Table S1: Interrupted time series of weekly EMS calls for mental health and overdose from January 2019 to July 2020, using negative binomial regression offset by total population, for regions in the UK and Canada

		East Midlands Region, UK	Niagara and Hamilton Regions, Canada
Outcome	Variable	Estimate [SE]	Estimate [SE]
Mental Health		n = 87,086*	n = 19,410*
	Intercept	**-8.88199 [0.02691]**	**-5.63500 [0.04596]**
	Per week	**0.00223 [0.00023]**	-0.00066 [0.00038]
	Lockdown	**0.11739 [0.04428]**	-0.07517 [0.07601]
	Lockdown trend	-0.00172 [0.00418]	0.00315 [0.00714]
Overdose		n = 64,832*	n = 11,619*
	Intercept	**-9.18762 [0.03036]**	**6.21481 [0.06800]**
	Per week	**0.00240 [0.00026]**	**0.00182 [0.00056]**
	Lockdown	**-0.55633 [0.05194]**	**-0.27632 [0.10919]**
	Lockdown trend	**0.03099 [0.00486]**	0.01451 [0.01030]

Notes: All models have been adjusted for seasonality (each month as a variable); Estimates statistically significant at $p < 0.05$ are indicated in bold; *total attendances in study period for each outcome

Table S2: For each sex and age category strata, an interrupted time series of weekly EMS calls for mental health from January 2019 to July 2020, using negative binomial regression offset by total population of subgroup, for regions in the UK and in Canada

		East Midlands Region, UK	Niagara and Hamilton Regions, Canada
Strata	Variable	Estimate [SE]	Estimate [SE]
Female		n = 50,098*	n = 10,829*
	Intercept	**-6.41376 [0.02799]**	**-1.38300 [0.05177]**
	Per week	**0.00243 [0.00024]**	-0.00001 [0.00043]
	Lockdown	**0.09583 [0.04545]**	0.01970 [0.08657]
	Lockdown trend	-0.00187 [0.00428]	-0.00805 [0.00813]
Male		n = 36,515*	n = 8,581*
	Intercept	**-9.09677 [0.03231]**	**-1.55274 [0.05365]**
	Per week	**0.00196 [0.00027]**	-0.00012 [0.00043]
	Lockdown	**0.15094 [0.05215]**	**-0.18591 [0.08571]**
	Lockdown trend	-0.00168 [0.00489]	**0.01719 [0.00797]**
<18 years		n = 4,239*	n = 1,572*
	Intercept	**-10.20282 [0.06694]**	-0.64919 [0.12505]
	Per week	**0.00286 [0.00058]**	-0.00090 [0.00100]
	Lockdown	**-0.31823 [0.11609]**	-0.37422 [0.21729]
	Lockdown trend	0.02060 [0.01069]	0.02401 [0.02068]
18-44 years		n = 39,648*	n = 10,382*
	Intercept	**-8.58670 [0.03366]**	**0.91736 [0.05066]**
	Per week	**0.00273 [0.00029]**	0.00026 [0.00042]
	Lockdown	0.09249 [0.05433]	-0.03127 [0.08358]

	Lockdown trend	-0.00555 [0.00511]	-0.00331 [0.00780]
45-65 years		n = 22,970*	n = 4,764*
	Intercept	**-8.93221 [0.03603]**	**0.56210 [0.07178]**
	Per week	**0.00216 [0.00031]**	-0.00002 [0.00058]
	Lockdown	**0.22702 [0.05680]**	-0.09937 [0.11530]
	Lockdown trend	-0.00872 [0.00534]	0.01212 [0.01071]
>65 years		n = 20,229*	n = 2,692*
	Intercept	**-8.64553 [0.03366]**	**0.65495 [0.08263]**
	Per week	**0.00117 [0.00029]**	-0.00081 [0.00066]
	Lockdown	**0.11159 [0.05433]**	-0.06335 [0.13371]
	Lockdown trend	**0.01107 [0.00508]**	0.00238 [0.01260]

Notes: All models have been adjusted for seasonality (each month as a variable); Estimates statistically significant at $p < 0.05$ are indicated in bold; *total attendances in study period for each strata

Table S3: For each sex and age category strata, an interrupted time series of weekly EMS calls for overdose from January 2019 to July 2020 using negative Binomial regression offset by total population, for regions in the UK and in Canada

		East Midlands Region, UK	Niagara and Hamilton Regions, Canada
Strata	Variable	Estimate [SE]	Estimate [SE]
Female		n = 29,939*	n =4,646*
	Intercept	**-6.91679 [0.03548]**	**-2.32028 [0.07615]**
	Per week	**0.00302 [0.00030]**	**0.00231 [0.00061]**
	Lockdown	**-0.62478 [0.06168]**	-0.09789 [0.11560]
	Lockdown trend	**0.03514 [0.00573]**	0.00577 [0.01085]
Male		n = 34,516*	n = 6,973*
	Intercept	**-9.16950 [0.03353]**	**-1.77332 [0.08298]**
	Per week	**0.00183 [0.00028]**	**0.00137 [0.00068]**
	Lockdown	**-0.49098 [0.05746]**	**-0.39374 [0.13325]**
	Lockdown trend	**0.02741 [0.00532]**	0.02095 [0.01255]
<18		n = 5,385*	n = 630*
	Intercept	**-10.01447 [0.05871]**	**-1.67502 [0.16952]**
	Per week	**0.00257 [0.00050]**	**-0.00284 [0.00133]**
	Lockdown	**-0.78563 [0.10820]**	-0.16779 [0.29054]
	Lockdown trend	**0.04929 [0.00975]**	0.00709 [0.02777]
18-44		n = 37,314*	n = 7,645*
	Intercept	**-8.65647 [0.03647]**	**0.46554 [0.07402]**
	Per week	**0.00235 [0.00031]**	**0.00197 [0.00060]**
	Lockdown	**-0.55932 [0.06296]**	**-0.35279 [0.11714]**

	Lockdown trend	**0.02988 [0.00587]**	0.01310 [0.01109]
45-65		n = 17,313*	n = 2,826*
	Intercept	**-9.25134 [0.03453]**	**0.13737 [0.10921]**
	Per week	**0.00213 [0.00029]**	**0.00274 [0.00090]**
	Lockdown	**-0.53919 [0.06029]**	-0.12434 [0.16900]
	Lockdown trend	**0.03052 [0.00546]**	0.01920 [0.01568]
>65		n = 4,820*	n = 518*
	Intercept	**-10.01568 [0.05934]**	-1.07290 [0.19175]
	Per week	**0.00327 [0.00050]**	0.00021 [0.00147]
	Lockdown	**-0.35730 [0.09831]**	-0.15615 [0.29888]
	Lockdown trend	**0.02398 [0.00889]**	-0.00381 [0.02815]

Notes: All models have been adjusted for seasonality (each month as a variable); Estimates statistically significant at $p < 0.05$ are indicated in bold; *total attendances in study period for each strata
