# Supplementary figures and images for: Ambulance Services Attendance for Mental Health and Overdose Before and During COVID-19 in Canada and the United Kingdom: Interrupted Time Series Study
Source: JMIR Public Health Surveill. 2024 May 10;10:e46029. doi: 10.2196/46029 (PMC11090162; doi:10.2196/46029)

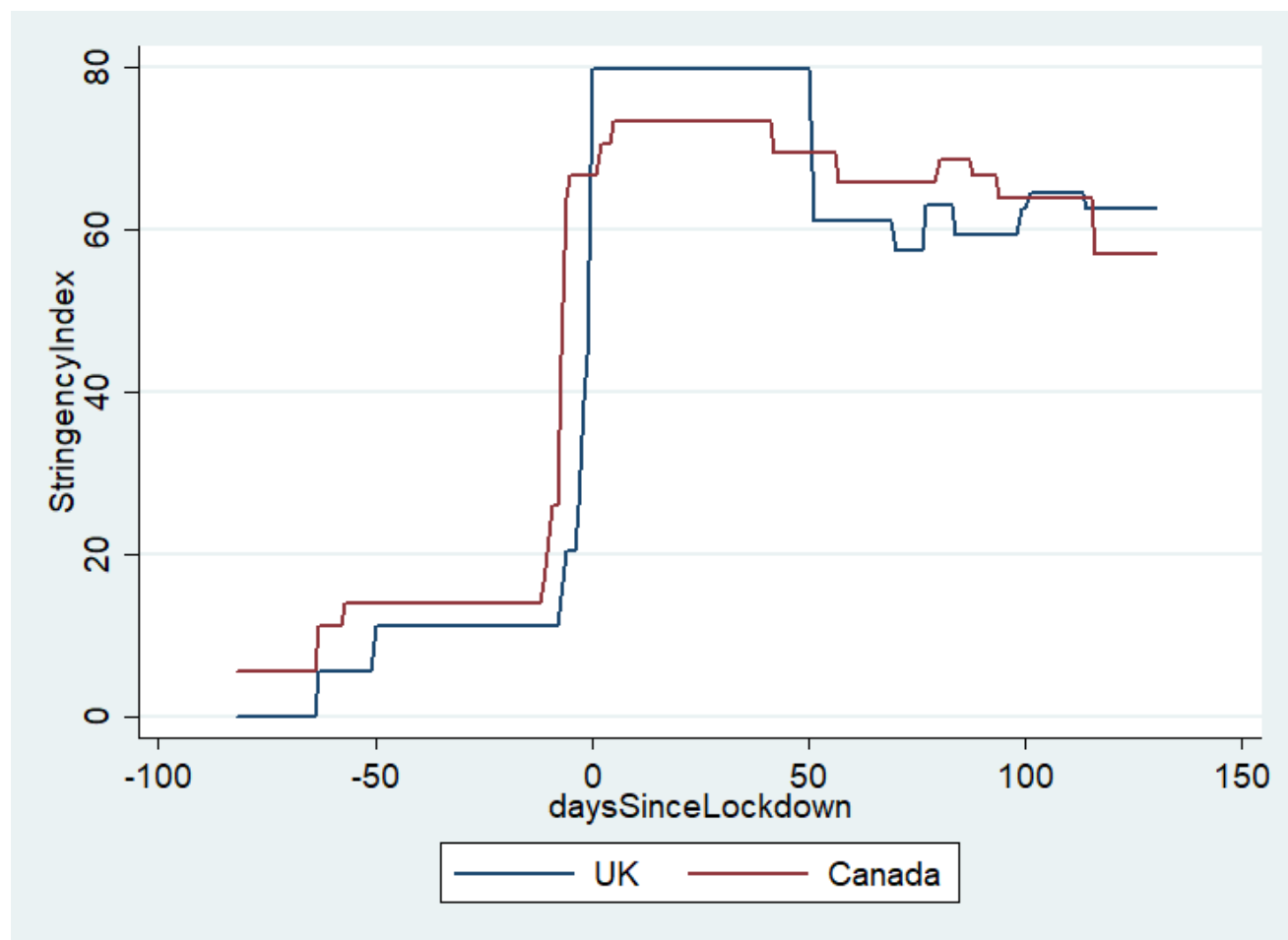

Supplement: Multimedia Appendix 2 [file publichealth_v10i1e46029_app2.pdf]
